# Suitability changes of *Citrus medica* L. var. *sarcodactylis* Swingle, a medicine-food plants affected by climate warming using the optimized MaxEnt model

**DOI:** 10.1371/journal.pone.0282659

**Published:** 2023-03-31

**Authors:** Yanli Xia, Muhammad Kazim, Muhammad Nabeel Nasir, Yuxia Yang, Qiang Li, Ting Li, Shiliang Xu, Yihe Wang, Xuchen Fan, Jinpeng Zhao, Rulin Wang

**Affiliations:** 1 School of Food and Bioengineering, Chengdu University, Chengdu, 610106, PR China; 2 Medical Officer THQ Hospital Kalur Kot, District BHAKKAR, Punjab, Pakistan; 3 Health Department Punjab, Punjab, Pakistan; 4 Sichuan Provincial Key Laboratory of Quality and Innovation Research of Chinese Materia Medica, Sichuan Academy of Traditional Chinese Medicine Sciences, Chengdu, 610041, PR China; 5 Zigong Meteorological Bureau, Zigong, 64300, PR China; 6 Sichuan Provincial Rural Economic Information Center, Chengdu, 610072, PR China; 7 Water-Saving Agriculture in Southern Hill Area Key Laboratory of Sichuan Province, Chengdu, 610066, PR China; Sakarya Uygulamali Bilimler Universitesi, TURKEY

## Abstract

Climatic variables are important conditions for plant growth, development and reproduction. *Citrus medica* L. var. *sarcodactylis* Swingle (Rutaceae: *Citrus*) is one of the traditional bulk Chinese medicinal materials in China with the effects of bacteriostasis, anti-inflammatory, anti-oxidation, anti-cancer cells, regulating the immun. Analyzing the impact of climate change on geographical distribution of *C*. *medica* L. var. *sarcodactylis* can provide strong support for its production layout and agricultural zoning. In our paper, MaxEnt and ArcGIS were applied to simulate the suitable areas of *C*. *medica* L. var. *sarcodactylis* in China from the perspectives of bioclimate, soil, topographic factors and human activities, and the future climate scenarios generated by global climate models (GCMs) were selected to predict its suitable areas in 2050s and 2090s. Results showed that, 1) Under current climate condition, areas of the total, most, moderately and poorly suitable habitats of *C*. *medica* L. var. *sarcodactylis* in China were 177.36×10^4^ km^2^, 22.27×10^4^ km^2^, 51.96×10^4^ km^2^ and 103.13×10^4^ km^2^ respectively. The range of the most suitable habitat was the narrowest, which was located in the middle east of Sichuan, western Chongqing in the upstream of the Yangtze River Basin, southern Guizhou and western Guangxi in the upstream of the Pearl River Basin, central and southern Yunnan and Southeast Tibet in the Middle-Lower reaches of the Southwest River Basin and western Taiwan. 2) Under the future climate change scenarios, the total suitable area showed a significant increase trend in 2090s, and the change of most, moderately and poorly suitable habitats showed no obvious law. 3) Under SSP1-2.6, SSP2-4.5 and SSP5-8.5 scenarios, the centroid of the most suitable habitat of *C*. *medica* L. var. *sarcodactylis* would move to the northwest, southeast and southwest respectively.

## 1 Introduction

Climatic variables are conditions for plant growth, development and reproduction [1,2]. The distribution of plants depends largely on abiotic factors, such as precipitation, temperature, soil and altitude [3,4]. Studying the impact of climate change on plant distribution is helpful to agricultural production and biodiversity protection, and can promote the sustainability of ecosystem. The drastic change of climate will lead to the change of the original habitat of plants, and then affect their suitable range [5–7]. Scientific evidences show that climate events such as climate warming, precipitation pattern change and atmospheric CO_2_ concentration rise have made a significant influence on species diversity, genetic diversity and landscape diversity [8,9]. The formation of traditional Chinese medicine is closely related to climate, and climate change will affect its suitable growth areas [10,11]. Among the nearly 200 genuine medicinal materials in China, some genuine areas have remained stable, such as *Chaenomeles speciosa* and *Fritillaria cirrhosa*, while some have undergone corresponding changes, such as *Alisma orientale* and *Citrus aurtantium* [10].

The global climate change characterized by the increase of temperature and the change of precipitation makes the natural system, biological system and human health system oscillate [12–14]. Agricultural production, which relies heavily on natural resources, shows obvious vulnerability under climate change. This is because climate determines the production potential and yield of crops to a certain extent [2]. Temperature rise will increase the heat stress of most regions and crops in the world, and the instability of water stress due to precipitation fluctuation will pose a threat to crop production [15,16]. The rainstorm, flood, high temperature and drought disasters brought by climate change have a direct impact on agricultural production and pose a great challenge to people’s growing demand and desire for a better life [17,18]. It is of great significance for agricultural production, social production and lifestyle to analyze the spatiotemporal change trend and distribution characteristics of temperature and precipitation under the background of climate change [19,20]. Teixeira et al. [21] found through simulation that under the influence of changes in heat stress under the A1B emission scenario, the area of the world’s crop suitable areas will change from 2071 to 2100, and the change in the area of corn and wheat will be much larger than that of rice and soybean. Tang et al. [22] calculated the spatial-temporal distribution of reference crop evapotranspiration in the Huang-Huai-Hai Plain under the main climate scenarios in the future by using temperature and precipitation data. The results provide basic data support for scientific allocation of agricultural water resources and scientific response to the impact of climate change on agricultural production.

*Citrus medica* L. var. *sarcodactylis* Swingle (Rutaceae: *Citrus*) is one of the traditional bulk Chinese medicinal materials in China, which was first contained in the first TCM treatise Shengnong Bencao Jing (also Shennon, g’s Classic of Materia Medica) [23]. The roots, stems, leaves, flowers and fruits of *C*. *medica* L. var. *sarcodactylis* can be used as medicine, and have unique functions of curing and preventing diseases, nourishing health and prolonging life [24]. Modern scientific experiments have shown that *C*. *medica* L. var. *sarcodactylis* has the effects of bacteriostasis, anti-inflammatory, anti-oxidation, anti-cancer cells, regulating the immune system [25]. At present, it is mainly used in medicine, food and daily chemical industries [26]. *C*. *medica* L. var. *sarcodactylis* is native to India and widely cultivated in France, Italy, Germany, the United States and Southeast Asia [23]. The planting areas in China are mainly distributed in Guangdong, Fujian, Chongqing, Sichuan and Zhejiang, especially Jiangjin in Chongqing and Zhaoqing in Guangdong, which have the largest planting area and the highest yield [27].

In recent years, the maximum entropy model (MaxEnt) has been applied to the study of habitat zoning of various medicinal plants under climate change scenarios since its good prediction ability. She et al. used MaxEnt to simulate the suitable distribution of the important medicinal resource plant *Notopterygium incisum* in the Three Rivers Headwater Region of China in the present and 2050s, and believed that the results would be conducive to the scientific protection and rational utilization of its resources [28]. Zhang et al. selected MaxEnt to compare the changes of the suitable habitat of wild *Anemarrhena asphodeloides* in China under the future climate change scenarios, and providing a theoretical basis for the protection of this medicinal plant and the scientific and reasonable increase of planting area [29]. The above research proves that MaxEnt model can be well applied to the study of habitat suitability analysis of medicinal plants.

With the increase of government investment in the traditional Chinese medicine industry, the cultivation area of *C*. *medica* L. var. *sarcodactylis* has been expanding [23]. However, without the guidance of scientific planting zoning and development planning, farmers planted it in large areas in non optimal growth areas, resulting in a series of problem. Therefore, it is necessary to analyze the suitability of *C*. *medica* L. var. *sarcodactylis* in China. In this paper, MaxEnt and ArcGIS were used to simulate the suitable areas of *C*. *medica* L. var. *sarcodactylis* in China from the perspectives of bioclimate, soil, topographic factors and human activities, and the climate change scenarios generated by global climate models (GCMs) were selected to predict its suitable areas in 2050s (2041–2060) and 2090s (2081–2100). The main purposes of this study are: 1) to provide scientific guidance for the reasonable planning and high-quality cultivation of *C*. *medica* L. var. *sarcodactylis* in China. 3) to master the impact of climate change on the planting suitability of *C*. *medica* L. var. *sarcodactylis*.

## 2 Materials and methods

### 2.1 Occurrence data of *C*. *medica L*. *var*. *sarcodactylis*

**Fig 1:**
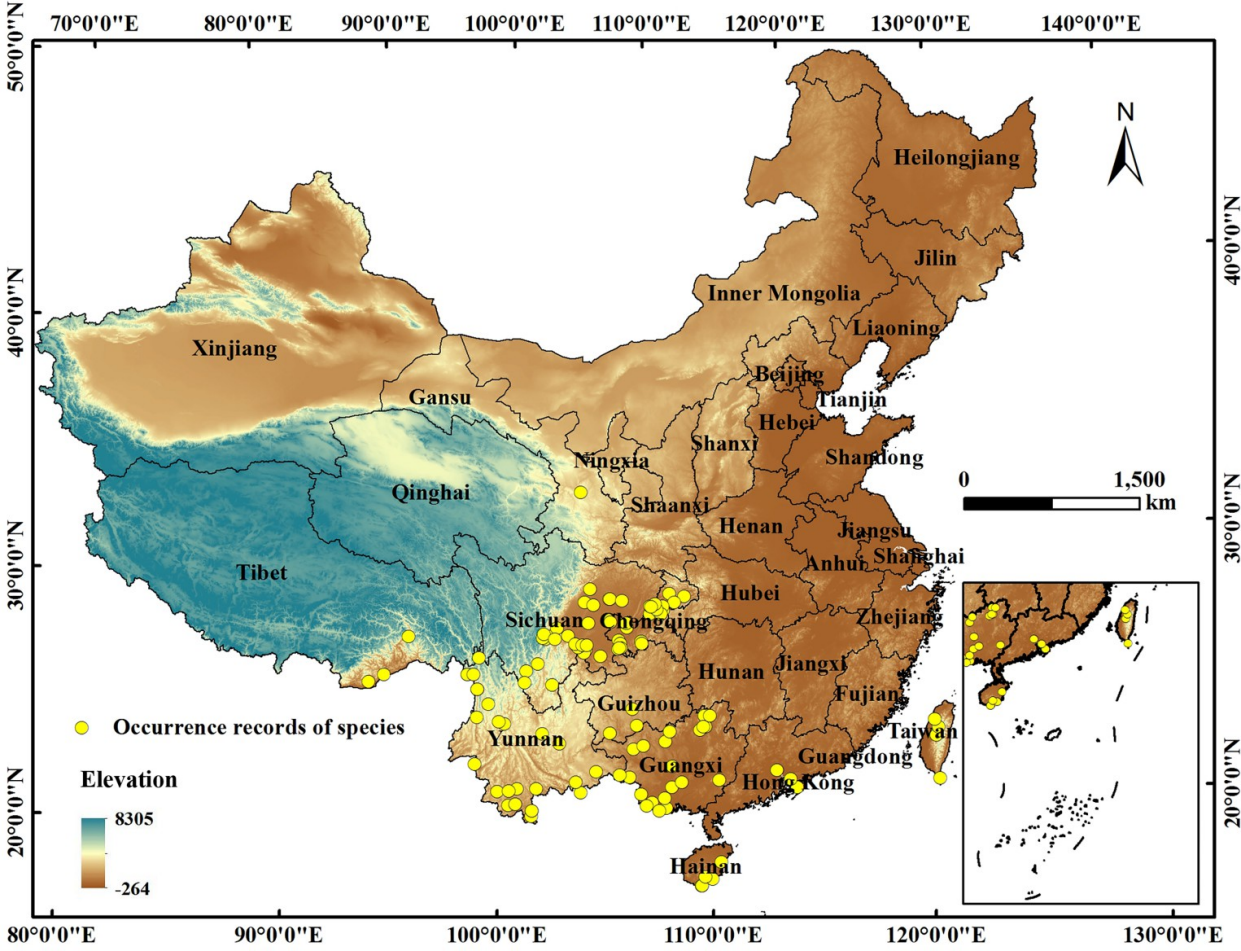
Occurrence records of *C*. *medica* L. var. *sarcodactylis*. The boundary was obtained from Natural Earth (http://www.naturalearthdata.com/). Based on the principle of national and territorial integrity, we have modified and adjusted the vector boundary.

Occurrence data of *C*. *medica* L. var. *sarcodactylis* were acquired from the Global Biodiversity Information Facility (GBIF, https://www.gbif.org/), the Chinese Virtual Herbarium (CVH, https://www.cvh.ac.cn/), field survey, and literature. Referring to the methods in literature [30,31], we processed the distribution records of *C*. *medica* L. var. *sarcodactylis*. Firstly, Baidu coordinate picking system (https://api.map.baidu.com/lbsapi/getpoint) was used to determine the longitude and latitude of records accurate to the town level. Second, ENMTOOL was applied to process the data to ensure the uniqueness of distribution points in each grid. After the above procedures, 123 records of *C*. *medica* L. var. *sarcodactylis* were retained for the establishment of MaxEnt (Fig 1).

### 2.2 Environmental data

In this study, 27 initial environment variables with a resolution of 2.5 arcminutes were selected (S1 Table). BCC-CSM2-MR (Beijing Climate Center Climate System Model) developed by China National Climate Center in CMIP6 which has been proved to be more suitable for China’s climate change characteristics was selected as the future climate model [32]. SSP1-2.6 (the lowly greenhouse gas emission scenario), SSP2-4.5 (the moderately greenhouse gas emission scenario) and SSP5-8.5 (the highly greenhouse gas emission scenario) in the Shared Socio-economic Pathways (SSPs) scenarios that can more scientifically describe future climate change were selected [33]. In order to reduce the influence of multi-collinearity among 19 bioclimatic variables, Pearson correlation analysis method was used [31,34]. Firstly, MaxEnt was used to calculate the percent contribution of 19 environmental variables, and the variables whose percent contribution rate was greater than 0 were retained (S1 Table). Thereafter, the Pearson’s coefficients between two variables with percent contribution greater than 0 corresponding to 123 occurrence data of *C*. *medica* L. var. *sarcodactylis* were analyzed using SPSS (S2 Table). Thirdly, by comparing the percentage contribution of the variables with the absolute value of the coefficient greater than 0.85, the higher one was retained. Finally, in addition to elevation, a total of 17 variables were selected to establish the prediction model of *C*. *medica* L. var. *sarcodactylis* (S1 Table).

### 2.3 Modelling process

MaxEnt software (Version 3.4.4, developed by Phillips et al. [39]) operation procedure was as follows: 1) The occurrence data of *C*. *medica* L. var. *sarcodactylis* in “CSV” format and the environmental variable in “ASC” format were imported into the "sample" and "environmental layers" data boxes respectively. 2) "Create response curves" and "Do jackknife to measure variable importance" were selected respectively to analyze the relationship between variables and presence probability of *C*. *medica* L. var. *sarcodactylis* and measure the importance of variables. 3) In the initial model, "Random test percentage" was set to 25%, while in the reconstructed model, "random seed" was selected, and the "replicates" was set to 10 [31,34,35]. 4) The Kuenm software package of R language was used to optimize the regularization multiplier (40, with values of 0.1~40) and feature combination (L, Q, P, T, H, LQ, LP, LT, LH, QP, QT, QH, PT, PH, TH, LQP, LQT, LQH, LPT, LPH, QPT, QPT, QPH, QTH, QTH, LQPT, LQPH, LQPH, LQTH, LQTH and LQPTH) of the model, and the optimal setting of the minimum information criterion AICc value (delta.AICc) among 1160 results was selected [36]. When the continuous prediction results are converted into the Boolean values of the "suitable habitat" and "unsuitable habitat", it is critical to select the appropriate threshold. In this study, the probability value P corresponding to the maximum sum of sensitivity and specificity was taken as the threshold value, that was, the probability of species existence p ≥ P was taken as the suitable hbitat, and p<P was taken as the unsuitable habitat. The suitable habitat was subdivided according to the following standards: poorl suitable habitat (P≤p<0.33), moderately suitable habitat (0.33≤p< 0.66) and most suitable habitat (p≥0.66) [6,37,38].

The area under the receiver operating characteristic (ROC) curve (AUC) was used to test the model prediction results. The evaluation criteria of AUC value were 0.5–0.6 (failure), 0.6–0.7 (poor), 0.7–0.8 (average), 0.8–0.9 (good), 0.9–1.0 (excellent) [39–41].

## 3 Results

### 3.1 Suitable habitat under current climate condition

**Fig 2:**
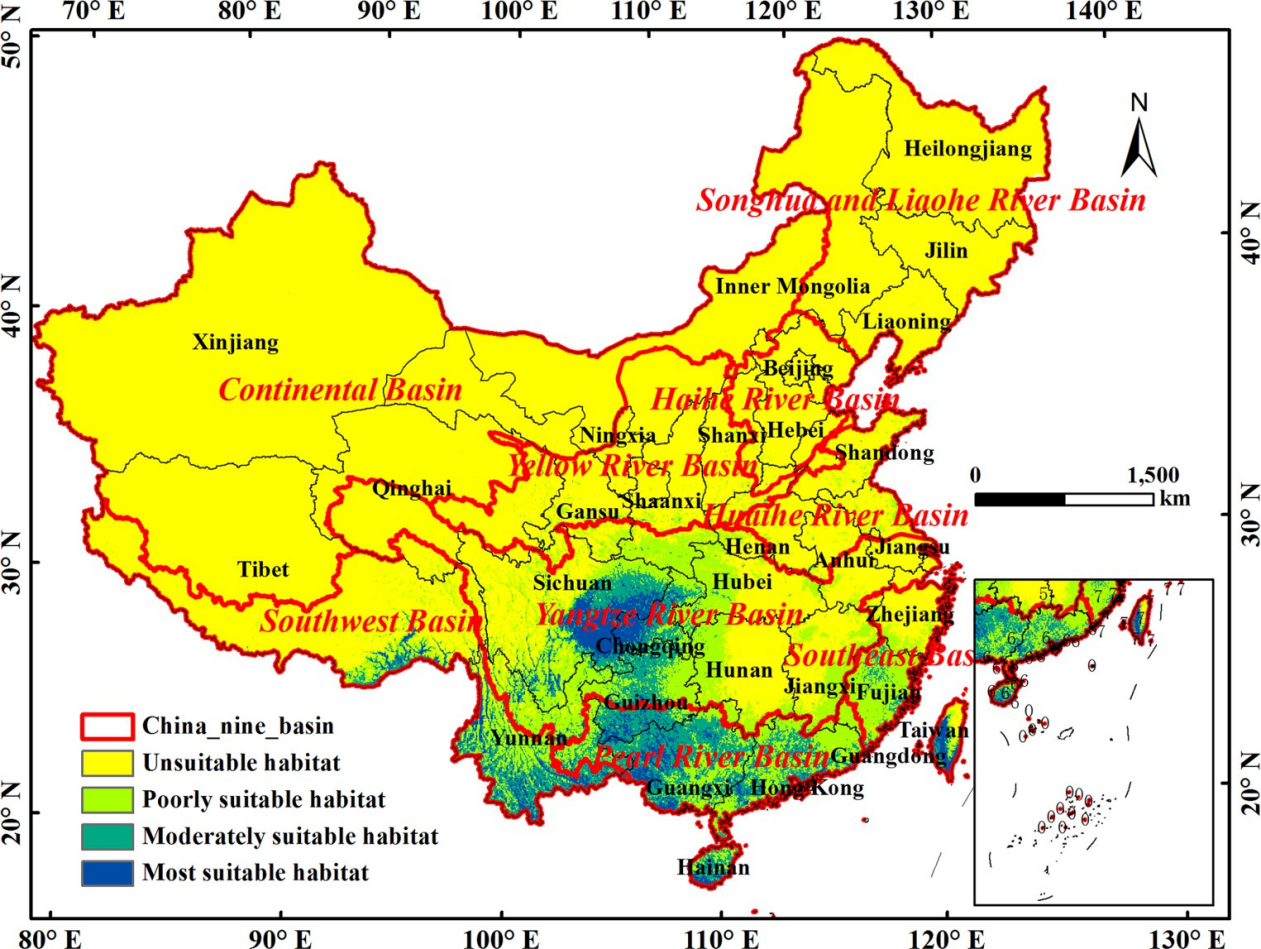
Suitable habitat simulated by MaxEnt under current climate situation. The boundary was obtained from Natural Earth (http://www.naturalearthdata.com/). Based on the principle of national and territorial integrity, we have modified and adjusted the vector boundary.

After reclassification and format conversion of the results output by MaxEnt, the area calculated by ArcGIS showed that the total suitable area of *C*. *medica* L. var. *sarcodactylis* in China was 177.36×10^4^ km^2^, accounting for 18.47% of the total area of the country, of which the distribution range of the most suitable habitat was the narrowest, with an area of 22.27×10^4^ km^2^, accounting for 12.55% of the total suitable habitat. Fig 2 showed that the most suitable habitat was located in the middle east of Sichuan, western Chongqing in the upstream of the Yangtze River and western Taiwan, southern Guizhou and western Guangxi in the upstream of the Pearl River, central and southern Yunnan and Southeast Tibet in the Middle-Lower reaches of the Southwest River. The moderate suitable habitat extended to the periphery along the most suitable habitat, with an area of 51.96×10^4^ km^2^, accounting for 29.3% of the total suitable habitat, mainly distributed in northeast Sichuan, central Chongqing and northern Guizhou in the upper reaches of the Yangtze River Basin, most of Guangxi in the upper reaches of the Pearl River Basin and central Guangdong in the lower reaches, southern Yunnan in the lower reaches of the Southwest River basin and most of Hainan (Fig 2). The poorly suitable habitat of *C*. *medica* L. var. *sarcodactylis* were mainly distributed in the northeast and south of Sichuan, the north of Chongqing, the south of Shaanxi, the west of Hubei, the west of Hunan, the northeast and northwest of Guizhou in the middle and upper reaches of the Yangtze River Basin, the east of Jiangxi in the lower reaches of the Yangtze River, most of Fujian in the southeast River Basin, most of Guangxi in the upper reaches of the Pearl River Basin and Guangdong in the lower reaches, southeast of Tibet and north central Yunnan in the lower reaches of the Southwest River basin, covering an area of 103.13×10^4^ km^2^, accounting for 58.15% of the total suitable habitat (Fig 2).

### 3.2 Future suitable habitat under climate change

**Fig 3:**
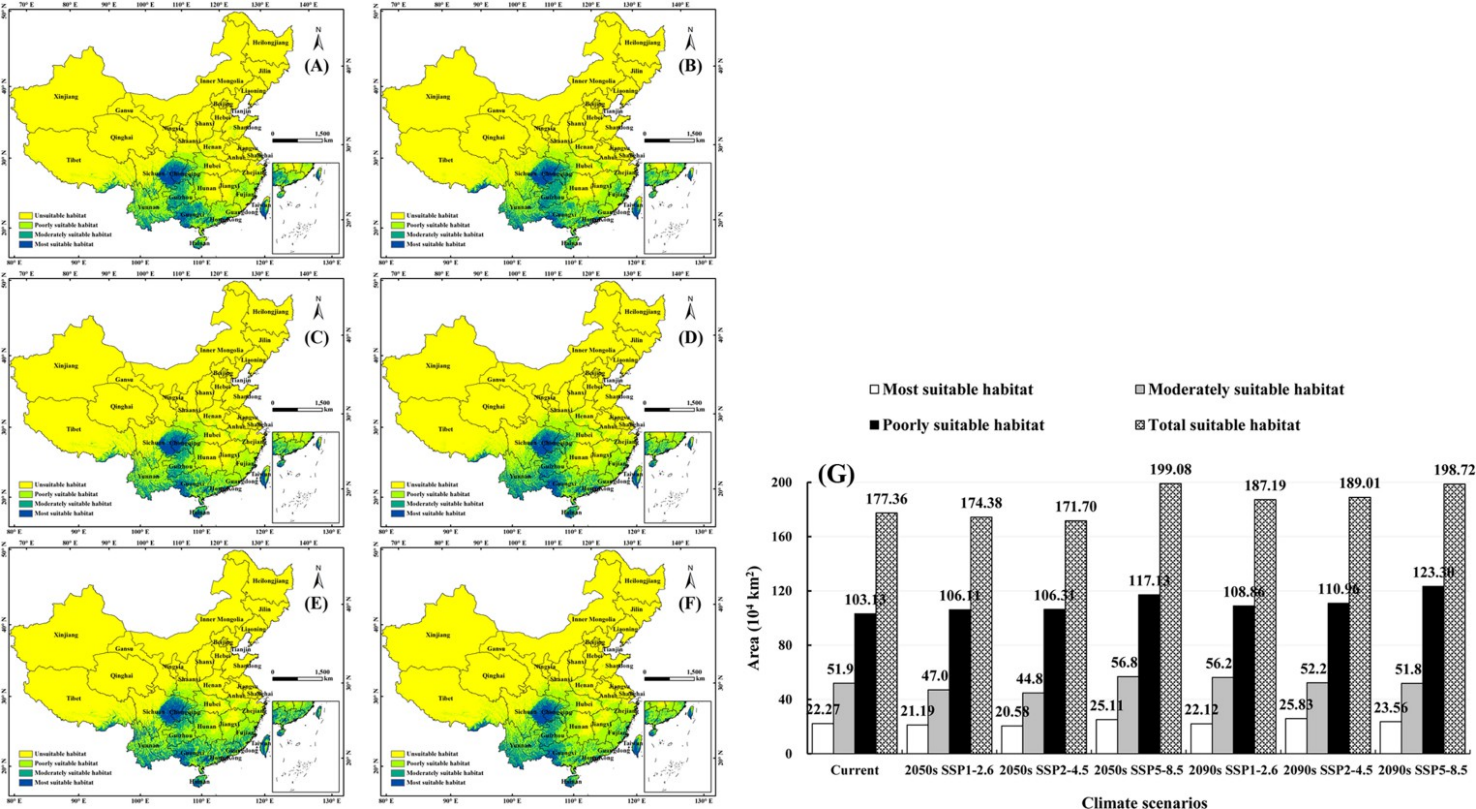
Suitable habitats simulated by MaxEnt under climate change scenarios. (A): 2050s, SSP1-2.6; (B): 2090s, SSP1-2.6; (C): 2050s, SSP2-4.5; (D): 2090s, SSP2-4.5; (E): 2050s, SSP5-8.5; (F): 2090s, SSP5-8.5; (G): Areas of suitable habitat under climate change scenarios. The boundary was obtained from Natural Earth (http://www.naturalearthdata.com/). Based on the principle of national and territorial integrity, we have modified and adjusted the vector boundary.

Under SSP1-2.6, the area of total suitable habitat was 174.38×10^4^ km^2^ (2050s) and 187.19×10^4^ km^2^ (2090s), which was decreased by 1.68% (2050s) and increased by 5.54% (2090s) compared with current, respectively. The areas of the most suitable habitats were 22.27×10^4^ km^2^ (2050s) and 22.12×10^4^ km^2^ (2090s), reducing by 4.85% and 0.65% compared with current situation, respectively. The areas of moderately suitable habitats were 47.08×10^4^ km^2^ (2050s) and 56.21×10^4^ km^2^ (2090s), which was decreased by 9.4% (2050s) and increased by 8.17% (2090s) compared with current. The areas of poorly suitable habitats were 106.11×10^4^ km^2^ (2050s) and 108.86×10^4^ km^2^ (2090s), which increased by 2.89% (2050s) and 5.55% (2090s) compared with current respectively (Fig 3A, 3B and 3G).

Under SSP2-4.5, the area of total suitable habitat was 171.7×10^4^ km^2^ (2050s) and 189.01×10^4^ km^2^ (2090s), which decreased by 3.19% (2050s) and increased by 6.57% (2090s) compared with current, respectively. The areas of most suitable habitats were 20.58×10^4^ km^2^ (2050s) and 25.83×10^4^ km^2^ (2090s), which decreased by 7.56% (2050s) and increased by 16% (2090s) compared with current, respectively. The areas of moderately suitable habitats were 44.81×10^4^ km^2^ (2050s) and 52.22×10^4^ km^2^ (2090s), which decreased by 13.77% (2050s) and increased by 0.5% (2090s) compared with current, respectively. The areas of poorly suitable habitats were 106.31×10^4^ km^2^ (2050s) and 110.96×10^4^ km^2^ (2090s), which increased by 3.08% (2050s) and 7.59% (2090s) compared with current, respectively (Fig 3C, 3D and 3G).

Under SSP5-8.5, the area of total suitable habitat was 199.08×10^4^ km^2^ (2050s) and 198.72×10^4^ km^2^ (2090s), which increased by 12.25% (2050s) and 12.04% (2090s) compared with current, respectively. The areas of most suitable habitats were 25.11×10^4^ km^2^ (2050s) and 23.56×10^4^ km^2^ (2090s), which increased by 12.79% (2050s) and 15.83% (2090s) compared with current, respectively. The areas of moderately suitable habitats were 56.84×10^4^ km^2^ (2050s) and 51.86×10^4^ km^2^ (2090s), which increased by 9.38% (2050s) and decreased by 0.2% (2090s) compared with current, respectively. The areas of poorly suitable habitats were 117.13×10^4^ km^2^ (2050s) and 123.3×10^4^ km^2^ (2090s), which increased by 13.57% (2050s) and 19.55% (2090s) compared with current, respectively (Fig 3E, 3F and 3G).

### 3.3 Changes in distribution of suitable habitats in the future

**Fig 4:**
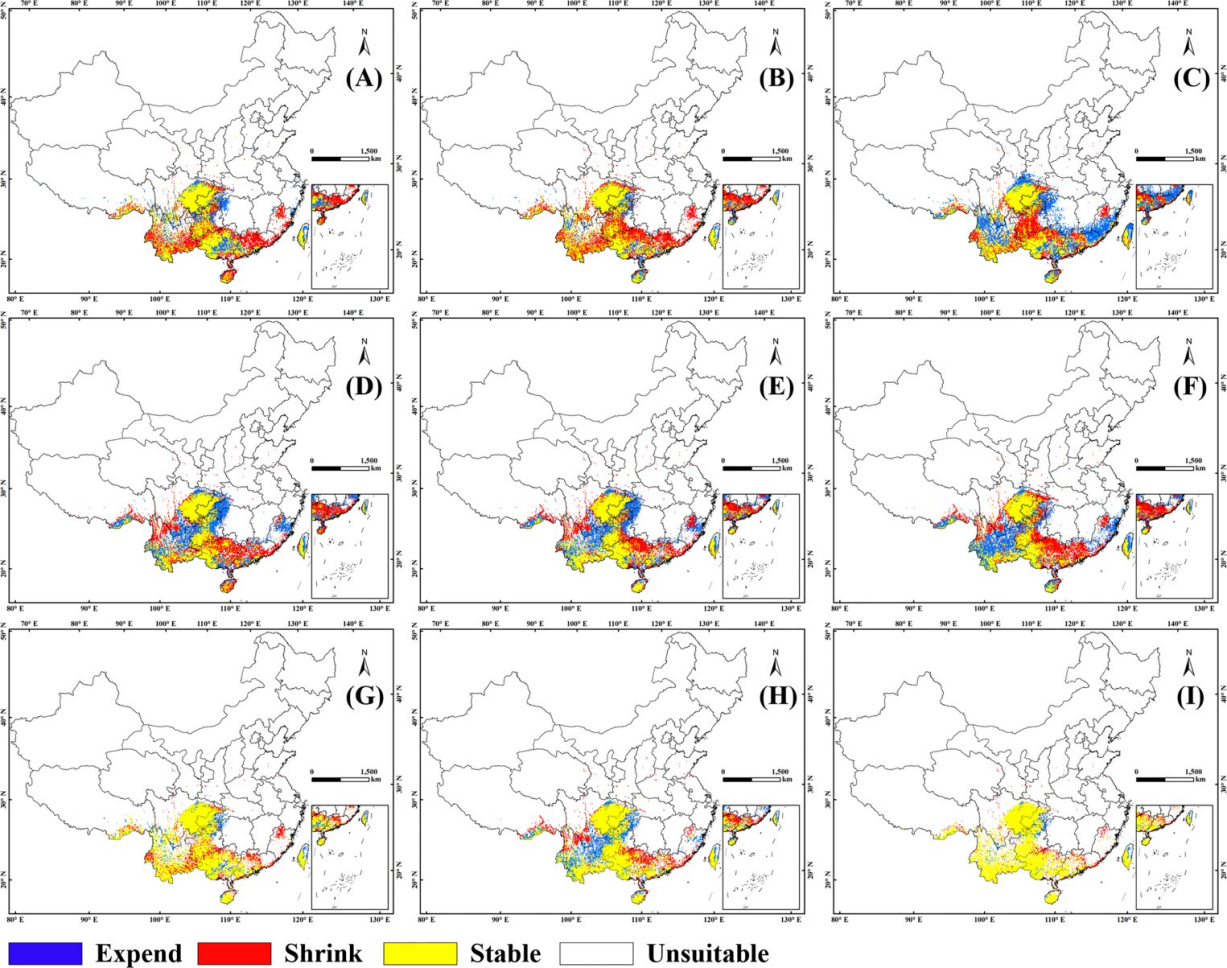
Changes in distribution of suitable habitats in the future. (A): 2050s, SSP1-2.6; (B): 2050s, SSP2-4.5; (C): 2050s, SSP5-8.5; (D): 2090s, SSP1-2.6; (E): 2090s, SSP2-4.5; (F): 2090s, SSP5-8.5; (G): 2050s; (H): 2090s; (I): 2050s~2090s. The boundary was obtained from Natural Earth (http://www.naturalearthdata.com/). Based on the principle of national and territorial integrity, we have modified and adjusted the vector boundary.

Fig 4A–4C showed the stable, expand and shrink area of the suitable habitat in 2050s compared with current. The area proportion of the stable habitat to the current was 89.66% (SSP1-2.6), 92.45% (SSP2-4.5) and 76.95% (SSP5-8.5) respectively, the area proportion of the shrink habitat to the current was 18.05% (SSP1-2.6), 19.07% (SSP2-4.5) and 15.26% (SSP5-8.5) respectively, and the area proportion of the expand habitat to the future was 10.34% (SSP1-2.6), 7.55% (SSP2-4.5) and 23.05% (SSP5-8.5) respectively. By overlie Fig 4A–4C in ArcGIS, the suitability change of the suitable habitat in 2050s was obtained (Fig 4G). The results showed that eastern Sichuan, Chongqing, most of Guizhou, southern Yunnan, most of Guangxi, western Guangdong and Hainan were identified as stable habitats for the growth and distribution of *C*. *medica* L. var. *Sarcodactylis* (Fig 4G). The expand habitat was dotted on the map with an area of 2.24×10^4^ km^2^, which was relatively concentrated only at the junction of Hubei, Chongqing, Hunan and Guizhou (Fig 4G). The area of the shrink habitat was 4.68×10^4^ km^2^, which was more obvious on the map as the northeast of Fujian, the middle of Guangdong, and the junction of Guizhou, Guangxi and Yunnan (Fig 4G).

**Fig 5:**
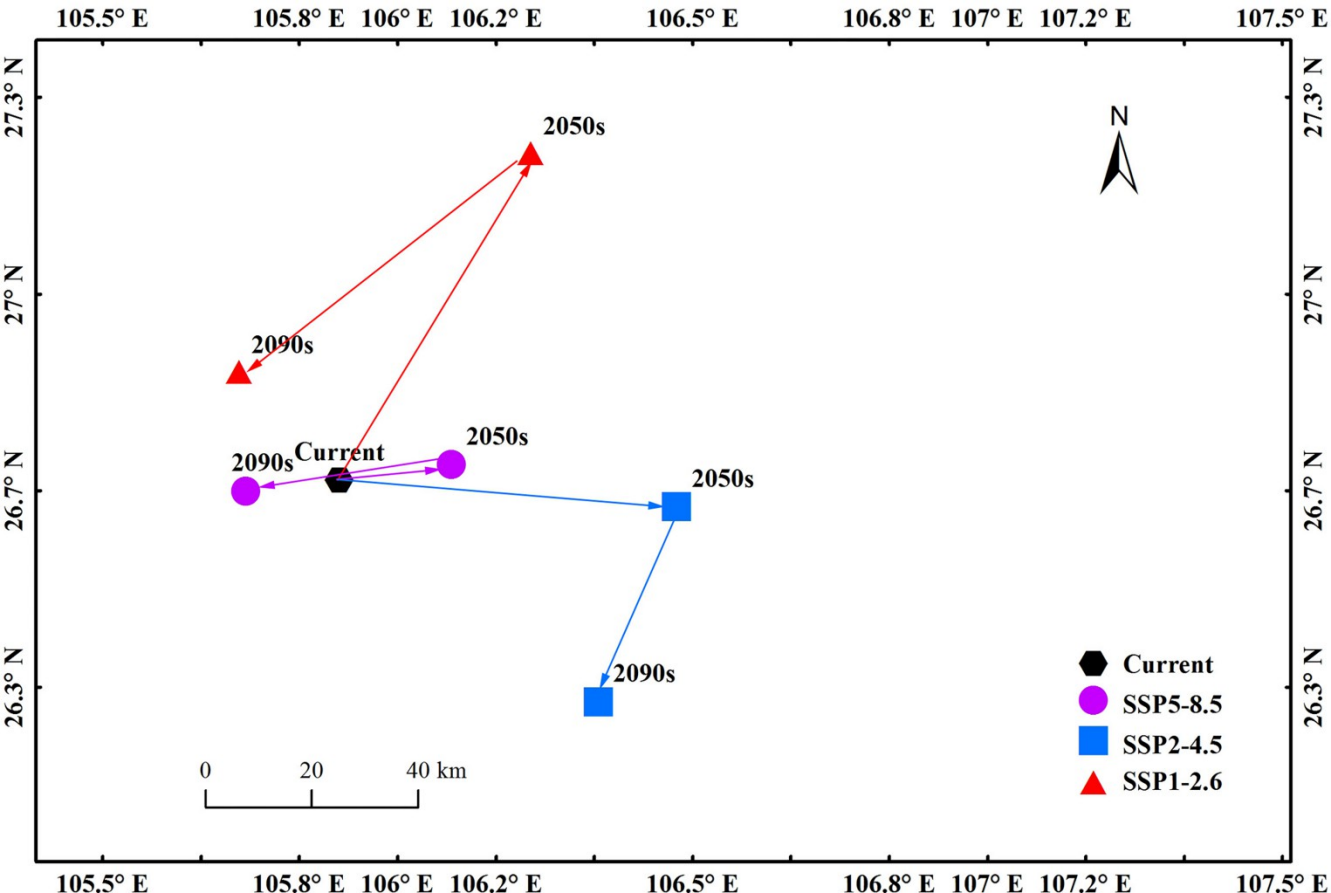
Trajectory changes of centroid in the future.

Fig 4D–4F showed the stable, expand and shrink area of the suitable habitat in 2090s compared with current. The area proportion of the stable habitat to the current was 84.19% (SSP1-2.6), 85.37% (SSP2-4.5) and 82% (SSP5-8.5) respectively, the area proportion of the shrink habitat to the current was 15.81% (SSP1-2.6), 14.62% (SSP2-4.5) and 18% (SSP5-8.5) respectively, and the area proportion of the expand habitat to the future was 20.05% (SSP1-2.6), 18.74% (SSP2-4.5) and 19% (SSP5-8.5) respectively. By overlie Fig 4D–4F in ArcGIS, the suitability change of the suitable habitat in 2090s was obtained (Fig 5H). The results showed that the distribution pattern of the stable habitat was basically consistent with that of 2050s (Fig 5H). Compared with 2050s, the expand habitat would increased significantly, especially at the junction of Sichuan, Yunnan and Guizhou. The shrink habitat was mainly located in northern Guangdong, southern Sichuan and northeastern Yunnan.

### 3.4 Trajectory changes of centroid in the future

Based on the centroid of the most suitable habitat under the current and future climate change scenarios, the movement trajectory were revealed as follows. 1) Under SSP1-2.6 scenario, the centroid would move from 105.9°E/26.68°N (current) to the northeast by 64.27 km to 106.22°E/27.23°N (2050s), and then to the southwest by 61.8 km to 105.73°E/26.86°N (2090s). From current to 2090s, the centroid would generally moved 24.97 km to the northwest (Fig 5). 2) Under SSP2-4.5 scenario, the centroid would move from 105.9°E/26.68°N (current) to the southeast by 57.43 km to 106.47°E/26.63°N (2050s), and then to the southwest by 35.64 km to 106.34°E/26.3°N (2090s). From current to 2090s, the centroid would generally moved 57.89 km to the southeast (Fig 5). 3) Under SSP5-8.5 scenario, the centroid would move from 105.9°E/26.68°N (current) to the northeast by 19.23 km to 106.09°E/26.7°N (2050s), and then to the southwest by 35.1 km to 105.74°E/26.66°N (2090s). From current to 2090s, the centroid would generally moved 15.88 km to the southwest (Fig 5).

### 3.5 Evaluation of MaxEnt models

Table 1 showed that the AUC values of the training data and test data of the MaxEnt model under current situation were 0.969±0.001 and 0.952±0.013 respectively. The AUC values of the training data of the future climate scenario models were 0.963±0.002–0.969±0.002 and that of the test data were 0.945±0.014–0.955±0.018.

**Table 1:** AUC values of models.

	Training data	Test data
Current	0.969±0.001	0.952±0.013
2050s, SSP1-2.6	0.969±0.002	0.95±0.016
2050s, SSP2-4.5	0.969±0.002	0.955±0.018
2050s, SSP5-8.5	0.964±0.001	0.948±0.019
2090s, SSP1-2.6	0.965±0.002	0.948±0.025
2090s, SSP2-4.5	0.965±0.002	0.95±0.017
2090s, SSP5-8.5	0.963±0.002	0.945±0.014

## 4 Discussion

Our simulation showed that the most suitable habitat of *C*. *medica* L. var. *Sarcodactylis* were distributed in the middle east of Sichuan, western Chongqing, southern Guizhou and western Guangxi, central and southern Yunnan and southeast Tibet, with an area of 22.27×10^4^ km^2^. *C*. *medica* L. var. *sarcodactylis* is native to India, mainly cultivated in China, with few wild species. With the cultivation and introduction, *C*. *medica* L. var. *sarcodactylis* has been widely planted in Sihui, Chaoshan, Yunfu and Yunan of Guangdong, Tianlin, Longlin, Lingle, Guanyang, Daxin and Yongfu of Guangxi, Kunming, Yuxi, Chuxiong, Xinping, Yimen, Eshan and Pu’er of Yunnan, Jiangjin, Yongchuan, Yunyang and Kai counties of Chongqing, Anxian, Hejiang, Yibin, Muchuan, Ya’an, Hongya, Jiajiang, Qianwei and Xingjing of Sichuan [27]. The above areas have a high degree of overlap with our simulation, indicating the accuracy of MaxEnt. According to the data, the actual planting area of *C*. *medica* L. var. *sarcodactylis* is far smaller than its suitable area. MaxEnt only simulates the probability of existence of *C*. *medica* L. var. *sarcodactylis* under the combination of dominant natural environmental variables, and does not involve production management and the impact on fruit yield and quality. In the actual production, not only the dominant natural resources, but also the social and economic factors such as labor, field management, production cost and market demand should be considered [23,42], so that the actual production area is smaller than the suitable habitat.

Climate change is one of the most important threats to global biodiversity in this century. Climate change may lead to changes in the future geographical distribution pattern of species, exacerbate the reduction of biodiversity and the loss of germplasm resources, and even accelerate the extinction rate of species [43,44]. Therefore, it is of great significance for species protection and sustainable utilization of resources to carry out research on the distribution pattern and change of species suitable areas in the context of climate change. Studying the response of plant distribution pattern to climate change and understanding the relationship between plant climate demand and geographical distribution is of great significance to reveal the history of species formation, migration and diffusion, and to put forward reasonable utilization strategies and planting zoning [45–47]. Global climate change is an important driving factor to change the geographical distribution pattern of species [48]. Some species benefit from climate change, and their distribution will be expanded, while the habitats of some species on the contrary will be reduced [37,38,49,50]. Our research showed that under the SSP1-2.6 and SSP2-4.5 in 2050s, the total suitable area of *C*. *medica* L. var. *sarcodactylis* changed slightly, while under other scenarios, the area increased significantly. This indicated that the future climate change would be favorable to *C*. *medica* L. var. *sarcodactylis* as a whole, and this expanding trend had also been found in other medicinal plants. Xu et al. predicted the distribution of *Thesium chinense* in China, and their results revealed that the area of the highly suitable area would increased gradually and expanded from the Yangtze River basin to the Yellow River Basin [51]. Zhao et al. analyzed the geographical distribution of Tibetan medicine *Lamiophlomis rotata* in the Qinghai Tibet Plateau, and the results showed that the expanded area of the suitable habitat caused by climate change was about twice the degraded area, and the area in all provinces showed an increasing trend [13]. The higher precipitation under the high concentration emission scenario can reduce the restriction of precipitation factors on the distribution of species and expand the suitable habitat of species. On the contrary, the increased precipitation under the low concentration emission scenario can not reduce the limit, but will reduce the available water for species to absorb with global warming [12,14,52]. This is consistent with the change law of the total suitable habitat of *C*. *medica* L. var. *sarcodactylis* under climate change. We speculated that the reduction of the total suitable area of *C*. *medica* L. var. *sarcodactylis* in 2050s may be due to the excessive drought caused by the temperature rise. While with the increase of years, rainfall gradually accumulated, making some areas suitable.

Trajectory analysis showed the centroid of the most suitable habitat of *C*. *medica* L. var. *sarcodactylis* would move to the northwest (SSP1-2.6), southeast (SSP2-4.5) and southwest (SSP5-8.5) respectively. This indicated that the impact of climate change on species distribution was uncertain, and the variation of temperature and precipitation under different scenarios may be the cause of this phenomenon. Xiang et al. found through simulation that under SSP1-2.6 scenario, the increasing trend of temperature rise rate and extreme precipitation rate in China from 2021 to 2100 was relatively flat, while under SSP2-4.5, SSP3-7.0 and SSP5-8.5 scenarios, they gradually increase with the increase of mode and time [53].

Since the 20th century, with the rapid growth of population and economy, human beings have had a great impact on their own living environment [54,55]. Climate change and human activities are considered to be the leading factors affecting the structure and function of terrestrial ecosystems [56,57]. Our analysis showed that human footprint was the key variables affecting the distribution of *C*. *medica* L. var. *sarcodactylis*, and the appropriate range was ≥ 6.99. *C*. *medica* L. var. *sarcodactylis* is mostly cultivated in China, and the impact of agricultural activities on its distribution cannot be ignored. Cutting, grafting and high-pressure propagation are the main propagation methods of *C*. *medica* L. var. *sarcodactylis* [25]. In recent years, with the continuous optimization of agricultural industrialization, the continuous adjustment of agricultural planting structure, and the influence of market regulation, the planting area of *C*. *medica* L. var. *sarcodactylis* has expanded [58,59]. However, Yue et al. [60] investigated the resources of *C*. *medica* L. var. *sarcodactylis* in Guangdong, and the results showed that due to the influence of production technology, market conditions, economic benefits and other factors, the planting area of *C*. *medica* L. var. *sarcodactylis* in Guangdong was shrinking year by year, and even sporadic cultivation was found in only 13 of the 40 sample plots visited. The above results all prove the importance of human activities to the cultivation and distribution of *C*. *medica* L. var. *sarcodactylis*.

## 5 Conclusions

Under current climate condition, areas of the total, most, moderately and poorly suitable habitats of *C*. *medica* L. var. *sarcodactylis* in China were 177.36×10^4^ km^2^, 22.27×10^4^ km^2^, 51.96×10^4^ km^2^ and 103.13×10^4^ km^2^ respectively. The range of the most suitable habitat was the narrowest, which was located in the middle east of Sichuan, western Chongqing in the upstream of the Yangtze River Basin, southern Guizhou and western Guangxi in the upstream of the Pearl River Basin, central and southern Yunnan and Southeast Tibet in the Middle-Lower reaches of the Southwest River Basin and western Taiwan. Under the future climate change scenarios, the total suitable area showed a significant increase trend in 2090s, and the changes of most, moderately and poorly suitable habitats showed no obvious law. Under SSP1-2.6, SSP2-4.5 and SSP5-8.5 scenarios, the centroid of the most suitable habitat would move to the northwest, southeast and southwest respectively. This study predicted the response of suitable habitats of *C*. *medica* L. var. *sarcodactylis* to climate change, which is of great significance for its planting division and resource protection.

## Supporting information

S1 Table Environmental variables used in this study. (DOCX)

S2 Table Pairwise Pearson’s correlation coefficients of climatic variables. (DOCX)

## References

[1] Gao W, Liu J, Xue Z, Zhang Y, Gao Z, Ni Y, et al. Geographical patterns and drivers of growth dynamics of Quercus variabilis. Forest Ecol Manag. 2018;429:256–66. 10.1016/j.foreco.2018.07.024.

[2] Lin BB, Egerer MH. Global social and environmental change drives the management and delivery of ecosystem services from urban gardens: A case study from Central Coast, California. Global Environmental Change. 2020;60:102006. 10.1016/j.gloenvcha.2019.102006.

[3] Mostofa MG, Rahman MM, Ghosh TK, Kabir AH, Abdelrahman M, Rahman Khan MA, et al. Potassium in plant physiological adaptation to abiotic stresses. Plant Physiol Bioch. 2022;186:279–89. 10.1016/j.plaphy.2022.07.011. 35932652

[4] Fairhurst SM, Jackson GE, Evans A, Cole LJ. The effect of pollination on the growth and reproduction of oilseed rape (Brassica napus). Basic Appl Ecol. 2022;63:164–74. 10.1016/j.baae.2022.06.007.

[5] Ashrafzadeh MR, Khosravi R, Mohammadi A, Naghipour AA, Khoshnamvand H, Haidarian M, et al. Modeling climate change impacts on the distribution of an endangered brown bear population in its critical habitat in Iran. Sci Total Environ. 2022;837:155753. doi: 10.1016/j.scitotenv.2022.155753 35526639

[6] Wei B, Wang R, Hou K, Wang X, Wu W. Predicting the current and future cultivation regions of Carthamus tinctorius L. using MaxEnt model under climate change in China. Glob Ecol Conserv. 2018;16:e477. 10.1016/j.gecco.2018.e00477.

[7] Burley H, Beaumont LJ, Ossola A, Baumgartner JB, Gallagher R, Laffan S, et al. Substantial declines in urban tree habitat predicted under climate change. Sci Total Environ. 2019;685:451–62. doi: 10.1016/j.scitotenv.2019.05.287 31176230

[8] Bhandari M, Jennison AV, Rathnayake IU, Huygens F. Evolution, distribution and genetics of atypical Vibrio cholerae–A review. Infection, Genetics and Evolution. 2021;89:104726. 10.1016/j.meegid.2021.104726. 33482361

[9] Cobo-Simón I, Méndez-Cea B, Jump AS, Seco J, Gallego FJ, Linares JC. Understanding genetic diversity of relict forests. Linking long-term isolation legacies and current habitat fragmentation in Abies pinsapo Boiss. Forest Ecol Manag. 2020;461:117947. 10.1016/j.foreco.2020.117947.

[10] Peng HS, Hao JD, Huang LQ. Effect of climate change on genuine medicinal materials producing areas during last 2000 years—Alisma orientale and Citrus aurtantium as examples. China Journal of Chinese Materia Medica. 2013;38(13):2218–22. 24079258

[11] Sun M, Zhang ZL. Research progress in medicinal plants response to climate change. Journal of Biology. 2015;32(5):84–8.

[12] Ren S, Chen X, Pan C. Temperature-precipitation background affects spatial heterogeneity of spring phenology responses to climate change in northern grasslands (30°N-55°N). Agr Forest Meteorol. 2022;315:108816. 10.1016/j.agrformet.2022.108816.

[13] Zhao WL, Chen HG, Yuan YY, Zhang J, Du T, Jin L. The impact of climate change on the distribution pattern of the suitable growing region for Tibetan medicine *Lamiophlomis rotata*. Acta Agrestia Sinica. 2021;29(5):956–64.

[14] Peltonen-Sainio P, Juvonen J, Korhonen N, Parkkila P, Sorvali J, Gregow H. Climate change, precipitation shifts and early summer drought: An irrigation tipping point for Finnish farmers? Climate Risk Management. 2021;33:100334. 10.1016/j.crm.2021.100334.

[15] Guzmán-Luna P, Mauricio-Iglesias M, Flysjö A, Hospido A. Analysing the interaction between the dairy sector and climate change from a life cycle perspective: A review. Trends Food Sci Tech. 2022;126:168–79. 10.1016/j.tifs.2021.09.001.

[16] Arshad M, Amjath-Babu TS, Aravindakshan S, Krupnik TJ, Toussaint V, Kächele H, et al. Climatic variability and thermal stress in Pakistan’s rice and wheat systems: A stochastic frontier and quantile regression analysis of economic efficiency. Ecol Indic. 2018;89:496–506. 10.1016/j.ecolind.2017.12.014.

[17] Hameed M, Ahmadalipour A, Moradkhani H. Drought and food security in the middle east: An analytical framework. Agr Forest Meteorol. 2020;281:107816. 10.1016/j.agrformet.2019.107816.

[18] Yang M, Mou Y, Meng Y, Liu S, Peng C, Zhou X. Modeling the effects of precipitation and temperature patterns on agricultural drought in China from 1949 to 2015. Sci Total Environ. 2020;711:135139. doi: 10.1016/j.scitotenv.2019.135139 32000347

[19] Abid M, Ali A, Rahut DB, Raza M, Mehdi M. Ex-ante and ex-post coping strategies for climatic shocks and adaptation determinants in rural Malawi. Climate Risk Management. 2020;27:100200. 10.1016/j.crm.2019.100200.

[20] Misra AK. Climate change and challenges of water and food security. International Journal of Sustainable Built Environment. 2014;3(1):153–65. 10.1016/j.ijsbe.2014.04.006.

[21] Teixeira EI, Fischer G, van Velthuizen H, Walter C, Ewert F. Global hot-spots of heat stress on agricultural crops due to climate change. Agr Forest Meteorol. 2013;170:206–15. 10.1016/j.agrformet.2011.09.002.

[22] Tang XP, Song N, Chen ZF, Wang JL. Spatial and temporal distribution of ET0 under main climate scenarios in future across Huang-Huai-Hai Plain. Transactions of the Chinese Society of Agricultural Engineering. 2016;32(14):168–76.

[23] Zhang SD, Yang HY, Zeng J, Li M. Research progress on Citri Sarcodactylis Fructus. China Journal of Traditional Chinese Medicine and Pharmacy. 2018;33(8):3510–4.

[24] Zhao XL. Research progress of physiologically active compounds of bergamot. Science and Technology of Food Industry. 2012;33(21):393–9.

[25] Peng B, Wen Y, Yu RM, Zhu JH. Advances in extraction, structure characterization and bioactivities of *Citrus medica* polysaccharide. Food and Drug. 2018;20(3):236–40.

[26] Huang XM, Deng X. Study on separation, purification and antioxidant activity of flavonoids from chuan bergamot. Chemical Research. 2017;28(6):730–9.

[27] Zhao YY, Zhang JY, Peng T, He PY, Kuang Y. Comparative analysis of the components of volatile oil in *Citrus medica* from different producing areas. China Pharmacy. 2020;31(4):423–8.

[28] She YD, Zhou HK, Zhang ZH, Ma L, Zhou BR, Song MH, et al. Suitable distribution of *Notopterygium incisum* in the Three Rivers Headwater region under climate change. Ecology and Environment Sciences. 2021;30(10):2033–41.

[29] Zhang ZY, Si MD, Wu M, Fan QF, Zhang SX, Zheng YG, et al. Prediction of distribution of potential suitable areas of wild *Anemarrhena asphodeloides* in China under future climate background. Journal of Chinese Medicinal Materials. 2022;45(1):37–42.

[30] Wang RL, Yang H, Wang MT, Zhang Z, Li Q. Predictions of potential geographical distribution of Diaphorina citri (Kuwayama) in China under climate change scenarios. Sci Rep-Uk. 2020;10(1):1–9. doi: 10.1038/s41598-020-66274-5 32513980 PMC7280263

[31] Liu L, Wang RL, Zhang YY, Mou QY, Gou YS, Liu K, et al. Simulation of potential suitable distribution of *Alnus cremastogyne* Burk. In China under climate change scenarios. Ecol Indic. 2021;133:108396.

[32] Zhou T, Chen Z, Zou L, Chen X, Zhang M. Development of climate and earth system models in China: past achievements and new CMIP6 results. Acta Meteorologica Sinica. 2020;78:332–50.

[33] Eyring V, S B, Meehl GA, Senior CA, Stevens B, Stouffer RJ, et al. Overview of the Coupled Model Intercomparison Project Phase 6 (CMIP6) experimental design and organization. Geosci Model Dev. 2016;9(5):1937–58.

[34] Yang J, Huang Y, Jiang X, Chen H, Liu M, Wang R. Potential geographical distribution of the edangred plant Isoetes under human activities using MaxEnt and GARP. Glob Ecol Conserv. 2022;38:e2186. 10.1016/j.gecco.2022.e02186.

[35] Guan L, Yang Y, Jiang P, Mou Q, Gou Y, Zhu X, et al. Potential distribution of Blumea balsamifera in China using MaxEnt and the ex situ conservation based on its effective components and fresh leaf yield. Environ Sci Pollut R. 2022;29:44003–19. doi: 10.1007/s11356-022-18953-1 35122650

[36] Zhao Z, Xiao N, Shen M, Li J. Comparison between optimized MaxEnt and random forest modeling in predicting potential distribution: A case study with Quasipaa boulengeri in China. Sci Total Environ. 2022;842:156867. doi: 10.1016/j.scitotenv.2022.156867 35752245

[37] Cheuk ML, Fischer GA. The impact of climate change on the distribution of Castanopsis (Fagaceae) species in south China and Indo-China region. Glob Ecol Conserv. 2021;26:e1388. doi: 10.1016/j.gecco.2020.e01388

[38] Gilani H, Arif Goheer M, Ahmad H, Hussain K. Under predicted climate change: Distribution and ecological niche modelling of six native tree species in Gilgit-Baltistan, Pakistan. Ecol Indic. 2020;111:106049. 10.1016/j.ecolind.2019.106049.

[39] Phillips SJ, Anderson RP, Schapire RE. Maximum entropy modeling of species geographic distributions. Ecol Model. 2006;190(3–4):231–59.

[40] Yang J, Huang Y, Jiang X, Chen H, Liu M, Wang R. Potential geographical distribution of the edangred plant Isoetes under human activities using MaxEnt and GARP. Glob Ecol Conserv. 2022;38:e2186. 10.1016/j.gecco.2022.e02186.

[41] Liu L, Guan LL, Zhao HX, Huang Y, Mou QY, Liu K, et al. Modeling habitat suitability of *Houttuynia cordata* Thunb (Ceercao) using MaxEnt under climate change in China. Ecol Inform. 2021;63(4):101324.

[42] Li CY, Yuan Z, She CJ, Bi JY. Research progress on chemical constituents and pharmacological actions of Citri Sarcodactylis Fructus. FOOD and Drug. 2022;24(2):187–93.

[43] Farooqi TJA, Irfan M, Portela R, Zhou X, Shulin P, Ali A. Global progress in climate change and biodiversity conservation research. Glob Ecol Conserv. 2022;38:e2272. 10.1016/j.gecco.2022.e02272.

[44] Lai Q, Hoffmann S, Jaeschke A, Beierkuhnlein C. Emerging spatial prioritization for biodiversity conservation indicated by climate change velocity. Ecol Indic. 2022;138:108829. 10.1016/j.ecolind.2022.108829.

[45] Tu W, Xiong Q, Qiu X, Zhang Y. Dynamics of invasive alien plant species in China under climate change scenarios. Ecol Indic. 2021;129:107919. 10.1016/j.ecolind.2021.107919.

[46] Ghehsareh Ardestani E, Heidari Ghahfarrokhi Z. Ensembpecies distribution modeling of Salvia hydrangea under future climate change scenarios in Central Zagros Mountains, Iran. Glob Ecol Conserv. 2021;26:e1488. 10.1016/j.gecco.2021.e01488.

[47] Gillison AN. Plant functional indicators of vegetation response to climate change, past present and future: II. Modal plant functional types as response indicators for present and future climates. Flora. 2019;254:31–58. 10.1016/j.flora.2019.04.001.

[48] Nascimbene J, Benesperi R, Casazza G, Chiarucci A, Giordani P. Range shifts of native and invasive trees exacerbate the impact of climate change on epiphyte distribution: The case of lung lichen and black locust in Italy. Sci Total Environ. 2020;735:139537. doi: 10.1016/j.scitotenv.2020.139537 32485454

[49] Shishir S, Mollah TH, Tsuyuzaki S, Wada N. Predicting the probable impact of climate change on the distribution of threatened Shorea robusta forest in Purbachal, Bangladesh. Glob Ecol Conserv. 2020;24:e1250. 10.1016/j.gecco.2020.e01250.

[50] Ribeiro-Souza P, Graipel ME, Astúa D, Vancine MH, Pires JSR. Effects of climate change on distribution and areas that protect two neotropical marsupials associated with aquatic environments. Ecol Inform. 2022;68:101570. 10.1016/j.ecoinf.2022.101570.

[51] Xu W, Du Q, Yan S, Cao Y, Liu X, Guan D, et al. Geographical distribution of As-hyperaccumulator Pteris vittata in China: Environmental factors and climate changes. Sci Total Environ. 2022;803:149864. doi: 10.1016/j.scitotenv.2021.149864 34500282

[52] Liu X, Lai Q, Yin S, Bao Y, Qing S, Bayarsaikhan S, et al. Exploring grassland ecosystem water use efficiency using indicators of precipitation and soil moisture across the Mongolian Plateau. Ecol Indic. 2022;142:109207. 10.1016/j.ecolind.2022.109207.

[53] Xiang JW, Zhang LP, Deng Y, She DX, Zhang Q. Projection and evaluation of extreme temperature and precipitation in major regions of China by CMIP6 models. Engineering Journal of Wuhan University. 2021;54(1):46–57.

[54] Zhang H, Wang Z. Human activities and natural geographical environment and their interactive effects on sudden geologic hazard: A perspective of macro-scale and spatial statistical analysis. Appl Geogr. 2022;143:102711. 10.1016/j.apgeog.2022.102711.

[55] Tischer C, Kirjavainen P, Matterne U, Tempes J, Willeke K, Keil T, et al. Interplay between natural environment, human microbiota and immune system: A scoping review of interventions and future perspectives towards allergy prevention. Sci Total Environ. 2022;821:153422. doi: 10.1016/j.scitotenv.2022.153422 35090907

[56] Zhai T, Wang J, Fang Y, Qin Y, Huang L, Chen Y. Assessing ecological risks caused by human activities in rapid urbanization coastal areas: Towards an integrated approach to determining key areas of terrestrial-oceanic ecosystems preservation and restoration. Sci Total Environ. 2020;708:135153. doi: 10.1016/j.scitotenv.2019.135153 31810665

[57] Hughes KA, Convey P. The protection of Antarctic terrestrial ecosystems from inter- and intra-continental transfer of non-indigenous species by human activities: A review of current systems and practices. Global Environmental Change. 2010;20(1):96–112. 10.1016/j.gloenvcha.2009.09.005.

[58] Qin GM, Rong RW, Ming YG. Isolation and characterization of neolignan derivatives with hepatoprotective and neuroprotective activities from the fruits of *Citrus medica L*. *var*. *Sarcodactylis* Swingle. Bioorg Chem. 2021;107:104622. 33454508 10.1016/j.bioorg.2020.104622

[59] Wu K, Jin R, Bao X, Yu G, Yi F. Potential roles of essential oils from the flower, fruit and leaf of *Citrus medica* L. var. sarcodactylis in preventing spoilage of Chinese steamed bread. Food Biosci. 2021;43(3):101271.

[60] Yue L, Cheng QQ, Yang Q. The resource survey of *Citri sarcodactylis* Fructus. Guangdong Chemical Industry. 2018;45(11):34–6.

