# Supplementary material for: Suitability changes of Citrus medica L. var. sarcodactylis Swingle, a medicine-food plants affected by climate warming using the optimized MaxEnt model
Source: PLoS One. 2023 Mar 31;18(3):e0282659. doi: 10.1371/journal.pone.0282659 (PMC10065301; doi:10.1371/journal.pone.0282659)
Supplement: S1 Table — (DOCX) [file pone.0282659.s001.docx]

**S1 Table.** Environmental variables used in this study.

| Type | Variables | Description | Unit | Percent contribution of initial model | Selected  /Eliminated |
| --- | --- | --- | --- | --- | --- |
| Bioclimate | Bio1 | Annual Mean Temperature | ℃ | 3.7 | Selected |
|  | Bio2 | Mean Diurnal Range(Mean of monthly (max temp - min temp) | ℃ | 2.9 | Selected |
|  | Bio3 | Isothermality (BIO2/BIO7) (* 100) | / | 1.1 | Eliminated |
|  | Bio4 | Temperature Seasonality (standard deviation *100) | ℃ | 1.5 | Eliminated |
|  | Bio5 | Max Temperature of Warmest Month | ℃ | 3.4 | Selected |
|  | Bio6 | Min Temperature of Coldest Month | ℃ | 0.4 | Eliminated |
|  | Bio7 | Temperature Annual Range (BIO5-BIO6) | ℃ | 19.9 | Selected |
|  | Bio8 | Mean Temperature of Wettest Quarter | ℃ | 1.1 | Eliminated |
|  | Bio9 | Mean Temperature of Driest Quarter | ℃ | 0.4 | Eliminated |
|  | Bio10 | Mean Temperature of Warmest Quarter | ℃ | 0.7 | Eliminated |
|  | Bio11 | Mean Temperature of Coldest Quarter | ℃ | 3.1 | Eliminated |
|  | Bio12 | Annual Precipitation | mm | 30.1 | Selected |
|  | Bio13 | Precipitation of Wettest Month | mm | 0.2 | Eliminated |
|  | Bio14 | Precipitation of Driest Month | mm | 3.5 | Eliminated |
|  | Bio15 | Precipitation Seasonality (Coefficient of Variation) | / | 5.0 | Selected |
|  | Bio16 | Precipitation of Wettest Quarter | mm | 2.4 | Eliminated |
|  | Bio17 | Precipitation of Driest Quarter | mm | 13.9 | Selected |
|  | Bio18 | Precipitation of Warmest Quarter | mm | 1.2 | Eliminated |
|  | Bio19 | Precipitation of Coldest Quarter | mm | 5.5 | Eliminated |
| DEM | El | Elevation | m | / | Selected |
| Soil | PH | Potential of hydrogen | / | / | Selected |
|  | T-C | Topsoil organic carbon | % | / | Selected |
|  | T-sand | Topsoil sand fraction |  | / | Selected |
|  | USDA | Topsoil USDA texture classification | name | / | Selected |
|  | Depth | Reference soil depth | m | / | Selected |
| Radiation | UV-B3 | Mean UV-B of Highest Month | kJ/m^2^ | / | Selected |
| Human activities | Hf | Human footprint index | / | / | Selected |
