# Supplementary material for: Suitability changes of Citrus medica L. var. sarcodactylis Swingle, a medicine-food plants affected by climate warming using the optimized MaxEnt model
Source: PLoS One. 2023 Mar 31;18(3):e0282659. doi: 10.1371/journal.pone.0282659 (PMC10065301; doi:10.1371/journal.pone.0282659)
Supplement: S2 Table — (DOCX) [file pone.0282659.s002.docx]

**S2 Table.** Pairwise Pearson’s correlation coefficients of climatic variables.

|  | Bio1 | Bio2 | Bio3 | Bio4 | Bio6 | Bio7 | Bio8 | Bio11 | Bio13 | Bio14 | Bio15 | Bio16 | Bio18 |
| --- | --- | --- | --- | --- | --- | --- | --- | --- | --- | --- | --- | --- | --- |
| Bio2 | -0.59 |  |  |  |  |  |  |  |  |  |  |  |  |
| Bio3 | -0.45 | 0.70 |  |  |  |  |  |  |  |  |  |  |  |
| Bio4 | 0.20 | 0.34 | -0.30 |  |  |  |  |  |  |  |  |  |  |
| Bio6 | 0.86 | -0.82 | -0.38 | -0.31 |  |  |  |  |  |  |  |  |  |
| Bio7 | -0.03 | 0.63 | 0.01 | 0.94 | -0.53 |  |  |  |  |  |  |  |  |
| Bio8 | 0.89 | -0.32 | -0.44 | 0.54 | 0.56 | 0.35 |  |  |  |  |  |  |  |
| Bio11 | 0.91 | -0.72 | -0.32 | -0.22 | 0.98 | -0.42 | 0.66 |  |  |  |  |  |  |
| Bio13 | 0.67 | -0.31 | -0.19 | 0.24 | 0.52 | 0.09 | 0.59 | 0.57 |  |  |  |  |  |
| Bio14 | 0.59 | -0.54 | -0.37 | 0.00 | 0.62 | -0.19 | 0.39 | 0.60 | 0.66 |  |  |  |  |
| Bio15 | -0.35 | 0.71 | 0.34 | 0.51 | -0.67 | 0.67 | 0.02 | -0.56 | -0.15 | -0.62 |  |  |  |
| Bio16 | 0.65 | -0.36 | -0.08 | 0.03 | 0.59 | -0.09 | 0.47 | 0.62 | 0.96 | 0.70 | -0.28 |  |  |
| Bio18 | 0.58 | -0.29 | 0.03 | 0.00 | 0.53 | -0.08 | 0.47 | 0.58 | 0.91 | 0.62 | -0.15 | 0.94 |  |
| Bio19 | 0.62 | -0.53 | -0.38 | 0.03 | 0.63 | -0.16 | 0.40 | 0.61 | 0.69 | 0.97 | -0.67 | 0.71 | 0.58 |
